# Multiple Bound States in the Continuum in Deep‐Subwavelength Nonlocal Polar Dielectric Nanoparticles

**DOI:** 10.1002/nap2.70233

**Published:** 2026-08-07

**Authors:** Man Lin, Ye Zhang, Xiaofeng Xu, Fengchuan Xu, Yunqiao Yin, Jie Luo, Lei Gao

**Affiliations:** ^1^ School of Physical Science and Technology Soochow University Suzhou China; ^2^ Collaborative Innovation Center of Suzhou Nano Science and Technology & Jiangsu Key Laboratory of Frontier Materia Physics and Devices Soochow University Suzhou China; ^3^ Jiangsu Key Laboratory of Biophotonics & Suzhou Key Laboratory of Biophotonics & SZCU‐ITU International Joint MetaCenter for Advanced Photonics and Electronics Suzhou City University Suzhou China; ^4^ Jiangsu Physical Science Research Center Nanjing China; ^5^ Collaborative Innovation Center of Suzhou Nano Science and Technology Soochow University Suzhou China

**Keywords:** multiple BICs, nanoparticles, nonlocal effect, polar dielectric

## Abstract

The confinement of light in open deep‐subwavelength nanostructures for enhanced light–matter interactions is a long‐standing goal in nanophotonics. Bound states in the continuum (BICs) offer a promising route to achieve light confinement, but their performance in deep‐subwavelength nanostructures is often limited. Here, we demonstrate that spatial dispersion (nonlocality) in polar dielectric nanoparticles enables the formation of multiple BICs, offering unprecedented confinement of light at the nanoscale. Through combined analytical and numerical studies of deep‐subwavelength core–shell nanoparticles (radius ∼ $\lambda_{0}/500$), we show that nonlocal polar dielectric shell supports multiple longitudinal‐transverse hybrid modes, manifesting as narrow Fano resonances in scattering spectra. Interestingly, by tuning geometric parameters, the radiative damping of these modes can be completely suppressed forming a series of BICs. Importantly, unlike conventional BICs in local‐response nanoparticles that require extreme physical conditions, these nonlocality‐induced BICs emerge across a broad parameter space. Our findings establish polar dielectrics as a versatile platform for multiple BICs for extreme subwavelength confinement with potential applications in sensing, nonlinear optics, and nanolasing.

## Graphical abstract

Spatial dispersion in polar‐dielectric core–shell nanoparticles enable multiple deep‐subwavelength bound states in the continuum through longitudinal‐transverse hybrid modes. By suppressing radiative damping at finite geometrical parameters, these nonlocality‐induced BICs provide a compact platform for high Q light confinement, enhanced light–matter interactions, sensing, nonlinear optics, and nanolasing.

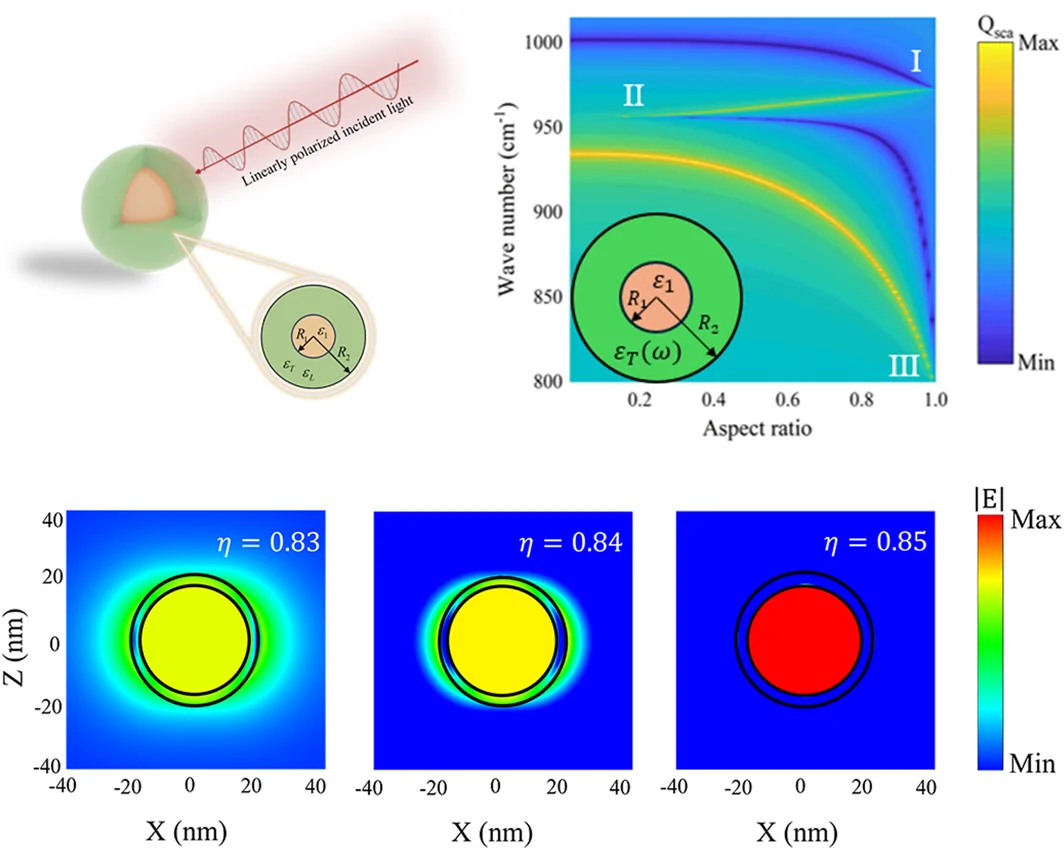

## 1 Introduction

Achieving highly confined electromagnetic fields has been a long‐standing goal in photonics. Traditional approaches typically rely on optical cavities or resonators formed by reflecting mirrors [1], photonic band‐gap materials [2, 3, 4], and ring structures [5, 6]. The emergence of bound states in the continuum (BICs) [7, 8, 9, 10, 11, 12, 13] has introduced a fundamentally different route to extreme light localization in open systems. BICs are special eigenstates embedded within the radiation continuum that nevertheless remain perfectly localized, exhibiting vanishing radiative linewidths and, in principle, infinite quality (Q) factors. Unlike traditional confinement mechanisms, BICs eliminate radiation loss through symmetry protection or destructive interference rather than physical barriers making them particularly attractive for nanoscale photonic systems. Originally proposed in quantum mechanics, the concept has since been extended to various classical wave platforms where ultrahigh‐Q BICs provide powerful opportunities for enhancing light–matter interactions [14]. In realistic structures, finite dimensions, material absorption, and other perturbations transform ideal BICs into Fano‐type resonances with large but finite Q factors commonly referred to as quasi‐BICs [15]. So far, various BIC manifestations have been demonstrated in waveguides [16, 17], metasurfaces [18, 19, 20, 21, 22, 23, 24], photonic crystals [25, 26], and resonators [27, 28].

Recent advances [29, 30, 31, 32, 33, 34, 35, 36] have extended the BIC concept to three‐dimensional (3D) open nanostructures using plasmonic materials, encompassing both wavelength‐scale [37] and subwavelength [38] geometries. In particular, wavelength‐scale plasmonic nanoparticles described within a local‐response framework have been shown to support light localization with infinitely large lifetimes [37]. Subsequently, embedded eigenstates (i.e., BICs) were demonstrated in subwavelength nanoparticles through precise material and geometric tuning again within a local‐response description [38]. Notably, the influence of spatial dispersion (i.e., nonlocality) in plasmonic nanoparticles has also been studied [39, 40]. For instance, wavelength‐scale nonlocal plasmonic core–shell nanoparticles were found to support multiple BICs arising from hydrodynamic nonlocality [41], and monopole BICs were demonstrated in nonlocal plasmonic nanoparticles [42]. These findings highlight that spatial dispersion can substantially modify the BIC landscape in plasmonic nanoparticles.

Polar dielectrics support surface phonon polaritons (SPhPs) within the Reststrahlen band, which lies between the longitudinal optical (LO) and transverse optical (TO) phonon frequencies [43]. Compared to plasmonic materials, polar dielectrics offer lower dissipation, sharper resonances and the ability to exhibit divergent permittivity [44]. Deep‐subwavelength polar dielectrics also exhibit pronounced nonlocal effects [44], similar to those in nanoscale metals, but with a different microscopic origin. In nanoscale metals, spatial dispersion arises from the compressibility and Fermi pressure of the conduction‐electron gas, leading to longitudinal electron‐density oscillations with a nonlocal parameter related to the Fermi velocity [45]. In contrast, nonlocality in polar dielectrics originates from spatial dispersion of ionic sublattice motion and optical phonons, giving rise to LO phonon modes [46]. These properties have enabled applications in sensing [47], imaging [48], energy harvesting [49], and time dynamics [50]. Despite these progresses, the role of nonlocality in enabling within BICs polar dielectric nanostructures, particularly at the deep‐subwavelength scale, remains largely unexplored.

In this work, we demonstrate that deep‐subwavelength polar dielectric nanoparticles (radius ∼ $\lambda_{0}/500$) support multiple BICs induced by nonlocality. We first analyze the BICs supported by such nanoparticles under a local‐response model and identify the corresponding conditions. We then incorporate the nonlocal optical response, focusing on how nonlocality reshapes the field distributions and establishes the interference conditions required for radiative cancellation. Through analytical modeling and numerical calculations, we reveal that nonlocality introduces new degrees of freedom at deeply subwavelength scales, enabling the system to host multiple BICs, achieve enhanced field localization. These findings establish polar dielectrics as a versatile platform for realizing multiple BICs, offering avenues for advanced nanophotonic applications, including ultrasensitive sensing, enhanced nonlinear optical processes, and nanolasing.

## 2 Results and Discussion

### 2.1 Theoretical Model

We investigate a spherical core–shell nanosphere consisting of a dielectric core (radius $R_{1}$) and a polar dielectric shell (outer radius $R_{2}$), as illustrated in Figure 1. The core is characterized by a constant relative permittivity $\varepsilon_{1}=1.5$. The polar dielectric shell is described by a transverse permittivity [44, 51]

(1) $$\varepsilon_{T}=\varepsilon_{\infty }\frac{\omega_{L}^{2}-\omega \;\left(\omega +i\gamma \right)}{\omega_{T}^{2}-\omega \;\left(\omega +i\gamma \right)},$$

where $\varepsilon_{\infty }$ is the high‐frequency dielectric constant; $\omega_{L}$ and $\omega_{T}$ represent the LO and TO phonon frequencies, respectively; γ is the damping constant. When the spatial dispersion is included, the electromagnetic response of the polar dielectric also involves longitudinal phonon modes. These modes are characterized by a wave vector $k_{L}$ satisfying the longitudinal phonon mode dispersion relation $\varepsilon_{L}\left(\omega ,k\right)=0$ [46]. Here, $\varepsilon_{L}$ is the longitudinal relative permittivity:

(2) $$\varepsilon_{L}=\varepsilon_{\infty }\frac{\omega_{L}^{2}-\omega \;\left(\omega +i\gamma \right)-\beta_{L}^{2}k^{2}}{\omega_{T}^{2}-\omega \;\left(\omega +i\gamma \right)-\beta_{L}^{2}k^{2}},$$

where $\beta_{L}$ is the LO phonon propagation velocity; k is the complex wave vector. This nonlocal continuum model provides a macroscopic description of spatially dispersive phonon dynamics and has been employed to describe the optical response of nanoscale polar dielectric structures, where it captures confined LO phonons and their coupling to electromagnetic fields beyond the local‐response approximation [44]. This framework is conceptually analogous to, yet physically distinct from, the hydrodynamic nonlocal description of the electron gas in nanoscale metals, which also incorporates spatial dispersion effects [45].

**FIGURE 1:**
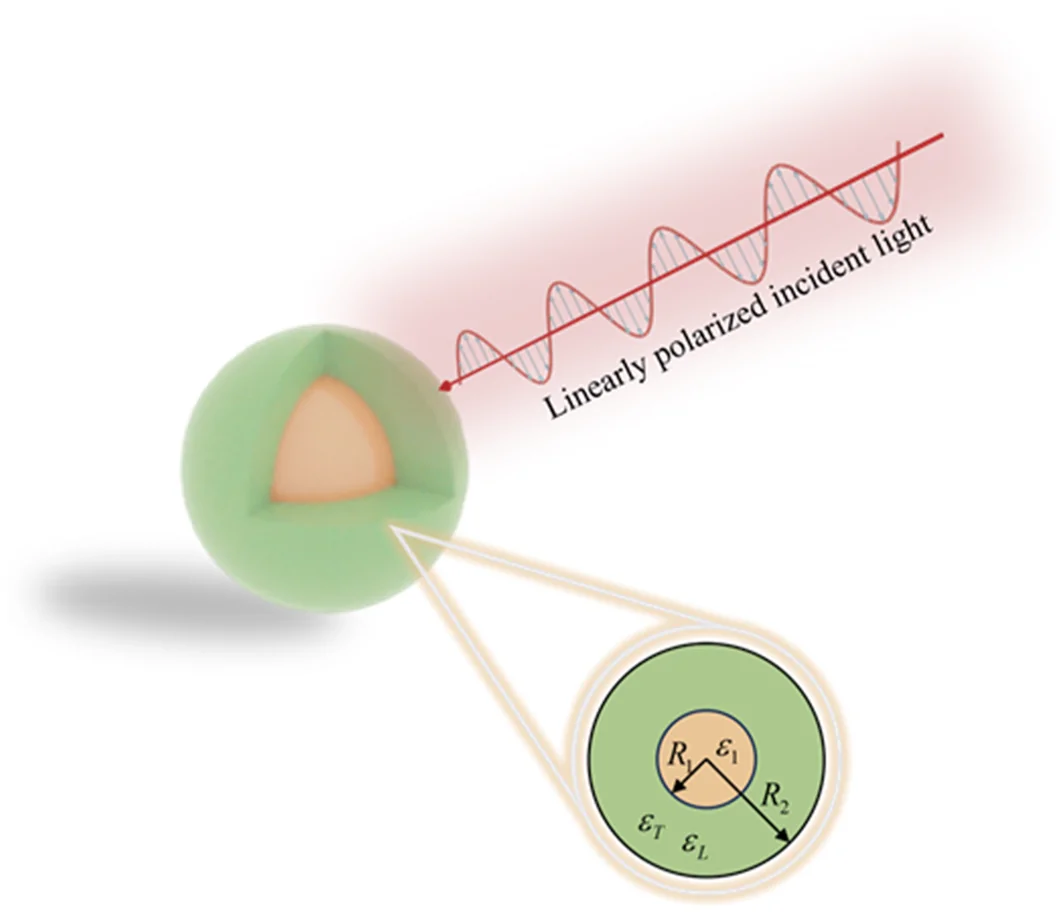
Schematic of a core–shell nanoparticle embedded in the host medium of relative permittivity $\varepsilon_{h}$ under plane‐wave illumination. The particle consists of a dielectric core (relative permittivity $\varepsilon_{1}$, radius $R_{1}$) coated by a polar dielectric shell (outer radius $R_{2}$).

We first analyze the optical scattering properties of the core–shell nanoparticle within a local‐response framework. For transverse magnetic (TM) modes, the electromagnetic fields in each region can be expressed in terms of spherical Bessel and Hankel functions according to Mie theory [52]. The incident and scattered electric fields are expressed as, respectively,

(3) $${\vec{E}}_{I}=\sum_{n=1}^{\infty }E_{n}\cdot \left\{-i\cdot \frac{1}{k_{h}}\nabla \times \nabla \times \left[\overset{\to }{r}\cdot j_{n}\left(k_{h}r\right)\cdot P_{n}^{\left(1\right)}\left(\cos\;\theta \right)\cos\;\phi \right]\right\},$$

and

(4) $${\vec{E}}_{S}=\sum_{n=1}^{\infty }E_{n}\cdot a_{n}\cdot \left\{i\cdot \frac{1}{k_{h}}\nabla \times \nabla \times \left[\overset{\to }{r}\cdot h_{n}\left(k_{h}r\right)\cdot P_{n}^{\left(1\right)}\left(\cos\;\theta \right)\cos\;\phi \right]\right\},$$

where $E_{n}=i^{n}E_{0}\left(2n+1\right)/\left(n\left(n+1\right)\right)$; $j_{n}\left(x\right)$ ($h_{n}\left(x\right)$) is the spherical Bessel (Hankel) function of the first kind; $P_{n}^{\left(1\right)}\left(\cos\;\theta \right)$ is the associated Legendre polynomial; $k_{h}=\left(\omega /c\right)\sqrt{\varepsilon_{h}}$ is the wave number in the host medium. For simplicity, we consider the nanosphere to be embedded in vacuum ($\varepsilon_{h}=1$).

The electric fields in the shell and core are written as, respectively,

(5) $${\vec{E}}_{T}=\sum_{n=1}^{\infty }E_{n}\cdot \left\{-i\cdot \frac{1}{k_{T}}\nabla \times \nabla \times \left[\overset{\to }{r}\cdot \left[g_{n}\cdot j_{n}\left(k_{T}r\right)+w_{n}\cdot y_{n}\left(k_{T}r\right)\right]\cdot P_{n}^{\left(1\right)}\left(\cos\;\theta \right)\cos\;\phi \right]\right\},$$

and

(6) $${\vec{E}}_{1}=\sum_{n=1}^{\infty }E_{n}\cdot d_{n}\cdot \left\{-i\cdot \frac{1}{k_{1}}\nabla \times \nabla \times \left[\overset{\to }{r}\cdot j_{n}\left(k_{1}r\right)\cdot P_{n}^{\left(1\right)}\left(\cos\;\theta \right)\cos\;\phi \right]\right\},$$

where $y_{n}\left(x\right)$ is the spherical Bessel function of the second kind; $k_{T}=\left(\omega /c\right)\sqrt{\varepsilon_{T}}$ is the wave number for the transverse electromagnetic modes; $k_{1}=\left(\omega /c\right)\sqrt{\varepsilon_{1}}$ is the wave number in the core.

The magnetic field in each region is obtained from the corresponding electric field through the relation $\vec{H}=\left(1/i\omega \mu \right)\nabla \times \vec{E}$. Then, considering the boundary continuity conditions at $r=R_{1}$ and $r=R_{2}$, the four unknown coefficients $\left(d_{n},g_{n},w_{n},a_{n}\right)$ can be solved. These coefficients can be obtained through solving the matrix equation $M_{\text{local}}\cdot x=0$ with $x={\left(d_{n},g_{n},w_{n},a_{n}\right)}^{T}$ and the matrix $M_{\text{local}}$ given by [37]

(7) $$M_{\text{local}}=\left(\begin{array}{cccc} {\left[j_{n}\left(k_{1}r\right)r\right]}_{r=R_{1}}^{\prime } & -{\left[j_{n}\left(k_{T}r\right)r\right]}_{r=R_{1}}^{\prime } & -{\left[y_{n}\left(k_{T}r\right)r\right]}_{r=R_{1}}^{\prime } & 0\\ \varepsilon_{1}j_{n}\left(k_{1}R_{1}\right) & -\varepsilon_{T}j_{n}\left(k_{T}R_{1}\right) & -\varepsilon_{T}y_{n}\left(k_{T}R_{1}\right) & 0\\ 0 & {\left[j_{n}\left(k_{T}r\right)r\right]}_{r=R_{2}}^{\prime } & {\left[y_{n}\left(k_{T}r\right)r\right]}_{r=R_{2}}^{\prime } & -{\left[h_{n}\left(k_{h}r\right)r\right]}_{r=R_{2}}^{\prime }\\ 0 & \varepsilon_{T}j_{n}\left(k_{T}R_{2}\right) & \varepsilon_{T}y_{n}\left(k_{T}R_{2}\right) & -\varepsilon_{h}h_{n}\left(k_{h}R_{2}\right) \end{array}\right).$$

The condition $\det\left({Μ}_{\text{local}}\right)=0$ defines the characteristic equation for the open spherical cavity. Its solutions correspond to the system's complex eigenfrequencies $\omega =\omega_{r}+i\omega_{i}$, where $\omega_{r}$ represents the eigenmode oscillation damping. In the lossless case, $\omega_{r}$ arises solely from radiation leakage into the far field. Notably, BICs emerge as special “embedded eigenmodes” with no radiation loss when the eigenfrequency becomes purely real (i.e., $\omega_{i}=0$) [37, 38], signifying the complete suppression of radiation loss.

Next, to incorporate spatial dispersion of the polar dielectric shell, the excitation of LO phonon modes is taken into consideration. The electric field associated with these modes is expressed as

(8) $${\vec{E}}_{L}=\sum_{n=1}^{\infty }E_{n}\cdot \frac{1}{k_{L}}\nabla \left\{\left[g_{n}^{L}\cdot j_{n}\left(k_{L}r\right)+w_{n}^{L}\cdot y_{n}\left(k_{L}r\right)\right]\cdot P_{n}^{\left(1\right)}\left(\cos\;\theta \right)\cos\;\phi \right\}.$$

For these modes, the magnetic field vanishes, that is, ${\vec{H}}_{L}=0$. The inclusion of longitudinal modes requires an additional boundary condition, i.e., the continuity of the relative ionic displacement [44]. By enforcing this condition concurrently with the standard electromagnetic boundary conditions, we obtain an extended linear system. In the nonlocal‐response framework, the governing matrix $M_{\text{nonlocal}}$ takes the form

(9) $$M_{\text{nonlocal}}=\left(\begin{array}{cccccc} -{\left[j_{n}\left(k_{1}r\right)r\right]}_{r=R_{1}}^{\prime } & {\left[j_{n}\left(k_{T}r\right)r\right]}_{r=R_{1}}^{\prime } & {\left[y_{n}\left(k_{T}r\right)r\right]}_{r=R_{1}}^{\prime } & j_{n}\left(k_{L}R_{1}\right) & y_{n}\left(k_{L}R_{1}\right) & 0\\ -\varepsilon_{1}j_{n}\left(k_{1}R_{1}\right) & \varepsilon_{T}j_{n}\left(k_{T}R_{1}\right) & \varepsilon_{T}y_{n}\left(k_{T}R_{1}\right) & 0 & 0 & 0\\ 0 & qj_{n}\left(k_{T}R_{1}\right) & qy_{n}\left(k_{T}R_{1}\right) & k_{L}R_{1}j_{n}^{\prime }\left(k_{L}R_{1}\right) & k_{L}R_{1}y_{n}^{\prime }\left(k_{L}R_{1}\right) & 0\\ 0 & qj_{n}\left(k_{T}R_{2}\right) & qy_{n}\left(k_{T}R_{2}\right) & k_{L}R_{2}j_{n}^{\prime }\left(k_{L}R_{2}\right) & k_{L}R_{2}y_{n}^{\prime }\left(k_{L}R_{2}\right) & 0\\ 0 & {\left[j_{n}\left(k_{T}r\right)r\right]}_{r=R_{2}}^{\prime } & {\left[y_{n}\left(k_{T}r\right)r\right]}_{r=R_{2}}^{\prime } & j_{n}\left(k_{L}R_{2}\right) & y_{n}\left(k_{L}R_{2}\right) & -{\left[h_{n}\left(k_{h}r\right)r\right]}_{r=R_{2}}^{\prime }\\ 0 & \varepsilon_{T}j_{n}\left(k_{T}R_{2}\right) & \varepsilon_{T}y_{n}\left(k_{T}R_{2}\right) & 0 & 0 & -h_{n}\left(k_{h}R_{2}\right) \end{array}\right),$$

which now encapsulates the contributions from the longitudinal response. Here, $q=n\left(n+1\right)\left(1-\frac{\varepsilon_{T}}{\varepsilon_{\infty }}\right)$. Solving the corresponding characteristic equation, $D\;\equiv \;\det\;\left(M_{\text{nonlocal}}\right)=0$, in the complex frequency plane allows us to identify the system's eigenmodes. Among these, BICs, arising from the interplay of transverse and longitudinal responses, can be identified as eigenmodes associated with purely real eigenfrequencies, satisfying $\omega_{i}=0$.

From the solved Mie coefficients, the scattering efficiency of the nanoparticle is obtained as

(10) $$Q_{sca}=\frac{2}{{k_{h}}^{2}{R_{2}}^{2}}\sum_{n=1}^{\infty }\left(2n+1\right){\left|a_{n}\right|}^{2}.$$

For deeply subwavelength particles, the scattering is dominated by the electric dipole term (n=1).

### 2.2 BICs in Local Nanoparticle

We now present the numerical and analytical results that elucidate the scattering characteristics and formation of BICs in the core–shell nanoparticle. The material of shell is chosen as 3C‐SiC [43, 49] with the following parameters: $\varepsilon_{\infty }=6.52$, $\omega_{L}=973{\text{cm}}^{-1}$, $\omega_{T}=796.1{\text{cm}}^{-1}$, and $\beta_{L}=15.39\times 10^{5}\text{cm}\cdot s^{-1}$. Material loss is neglected (γ=0), the outer radius is fixed at $R_{2}=20\;\text{nm}$, and the inner radius $R_{1}$ is tuned by varying the aspect ratio $\eta =R_{1}/R_{2}$. At the LO phonon frequency, the corresponding free‐space wavelength is $\lambda_{0}\;\approx \;10.3\;\mu m$, indicating that the core–shell nanoparticle with a radius $R_{2}∼\lambda_{0}/500$, operates in the deep‐subwavelength regime.

Figure 2a shows the scattering efficiency $Q_{sca}$, calculated from Equations (7) and (10), as a function of the aspect ratio η and frequency within the local‐response framework. Characteristic Fano resonance profiles are formed by the spectral interplay between scattering zeros (minima of $Q_{sca}\;\approx \;0$) and scattering resonances (maxima of $Q_{sca}$). For illustration, the scattering efficiency $Q_{sca}$ at a fixed aspect ratio of η=0.8 (as marked by the white dashed line in Figure 2a), is shown in Figure 2b where Fano line shapes are evident.

**FIGURE 2:**
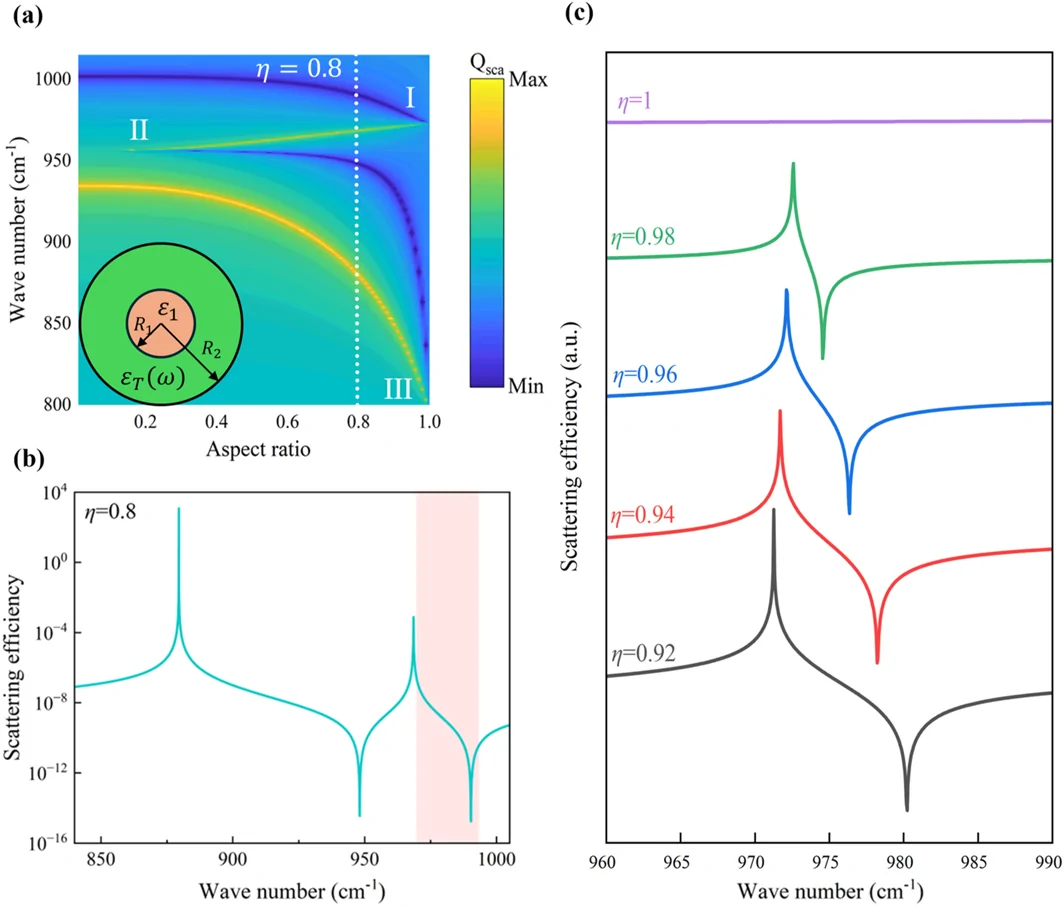
(a) Dependence of scattering efficiency $Q_{sca}$ on incident frequency ω and aspect ratio η for core–shell nanospheres. Three BICs are identified and labeled I, II, and III. (b) Scattering efficiency as a function of wavenumber with $R_{1}=16\;\text{nm}$ and η=0.8, corresponding to the vertical dashed line marked in (a). (c) Scattering efficiency $Q_{sca}$ for different η evaluated within the frequency range highlighted in the shaded region of (b).

Interestingly, by tuning the spectral positions of scattering zeros and resonances to approach each other, the Fano resonance profile sharpens and its Q factor increases. At the critical point of coalescence, a BIC with a diverging Q factor emerges. Three such BICs are identified and labeled I, II, and III in Figure 2a. To illustrate this evolution, Figure 2c focuses on the spectral range near BIC I as η is varied. We see that as η increases from 0.92 to 1, the Fano line linewidth progressively narrows, signifying a significant enhancement of the Q factor. When η=1, the scattering zero and resonance overlap completely, forming a BIC where the Q factor diverges.

We next examine the underlying mechanisms that give rise to these BICs. Each BIC corresponds to an extreme condition of the material or geometry: ε=0, η→1 for BIC I, $\varepsilon_{2}=-\varepsilon_{1}/2$, η→0 for BIC II, and ε=+∞, η→1 for BIC III. The BIC I arises in the limit of an infinitely thin epsilon‐near‐zero shell analogous to effects reported in plasmonic nanoparticles [37]. In this case, the electric field is strongly confined within the shell layer. Figure 3a–c show the electric‐field amplitude distributions for different η. When η=0.99, the field exhibits a distinct ring‐like localization inside the shell with strongly suppressed external radiation (Figure 3a). As η decreases, the electric field gradually leaks outward, demonstrating the transition from a BIC to a radiating quasi‐BIC (Figure 3b,c). The BIC II originates from the SPhP resonance at the core–shell interface under the condition $\varepsilon_{2}=-\varepsilon_{1}/2$ [38], in the limit where the core becomes vanishingly small. The field distributions in Figure 3d confirm that the mode is tightly localized inside the dielectric core with negligible field in the surrounding region. As η increases, the fields become less localized (Figure 3e,f).

**FIGURE 3:**
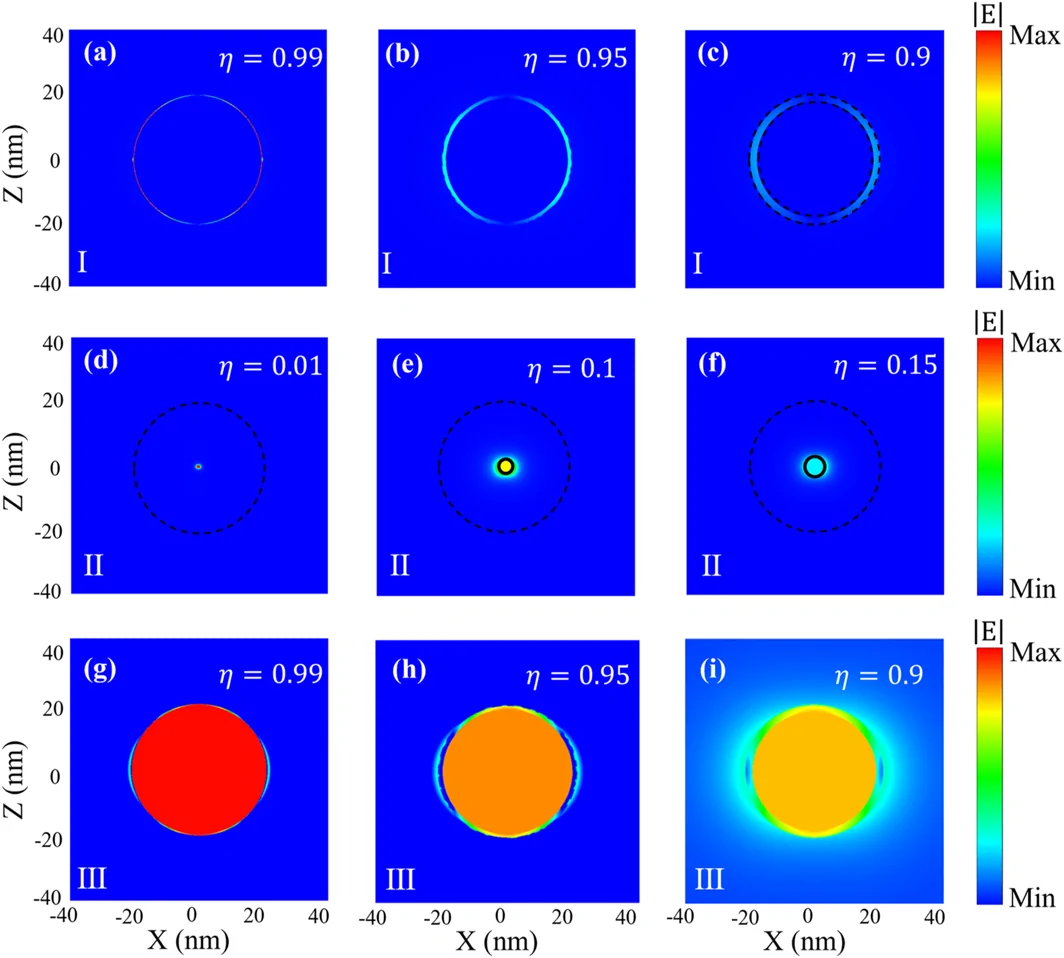
Evolution of the electric‐field amplitude distributions near BIC I (a–c), BIC II (d–f), and BIC III (g–i) for different η under the plane‐wave illumination.

We note that the BIC III is absent in previously proposed plasmonic nanoparticles [37, 38], as it is uniquely enabled by the Lorentz‐type dispersion of polar dielectrics. It occurs where the permittivity diverges (i.e., ε=+∞) near the TO phonon resonance, leading to a strong suppression of the internal electric field while maintaining pronounced interfacial polarization [44]. As shown in Figure 3g–i, the electric field in this case resembles typical electric dipole patterns, whereas the internal field is almost completely screened.

### 2.3 Multiple BICs in Nonlocal Nanoparticles

In the above analysis, the polar dielectric shell was treated within a local‐response framework where its permittivity is assumed independent of wavevector. At the nanoscale, however, nonlocal effects often become significant [44]. In this section, we incorporate the nonlocal response of the polar dielectric shell and investigate its role in generating multiple BICs.

Figure 4a presents the scattering efficiency $Q_{sca}$, calculated from Equations (9) and (10) for the nonlocal nanoparticle as a function of aspect ratio η and wave number. A key distinction from the local‐response case (see Figure 2a) is the appearance of numerous additional scattering zeros and resonances. In the local model, only three BICs arise from specific intersections between scattering zeros and resonances. The nonlocality, by introducing LO phonon modes within the polar dielectric shell, generates multiple scattering zeros and resonances and forms multiple narrow Fano resonances (Figure 4b). These Fano resonances arise from the interaction of the transverse and longitudinal modes in the core–shell nanoparticle. By tuning the geometry, the scattering zeros and resonances associated with these Fano resonances can be made to overlap, thereby forming a series of BICs. This phenomenon is absent in the local model, highlighting the additional degrees of freedom in realizing BICs enabled by the nonlocality. This is in contrast to nonlocal metallic structures [41, 42] where multiple BICs arise for frequencies $\omega >\omega_{p}$.

**FIGURE 4:**
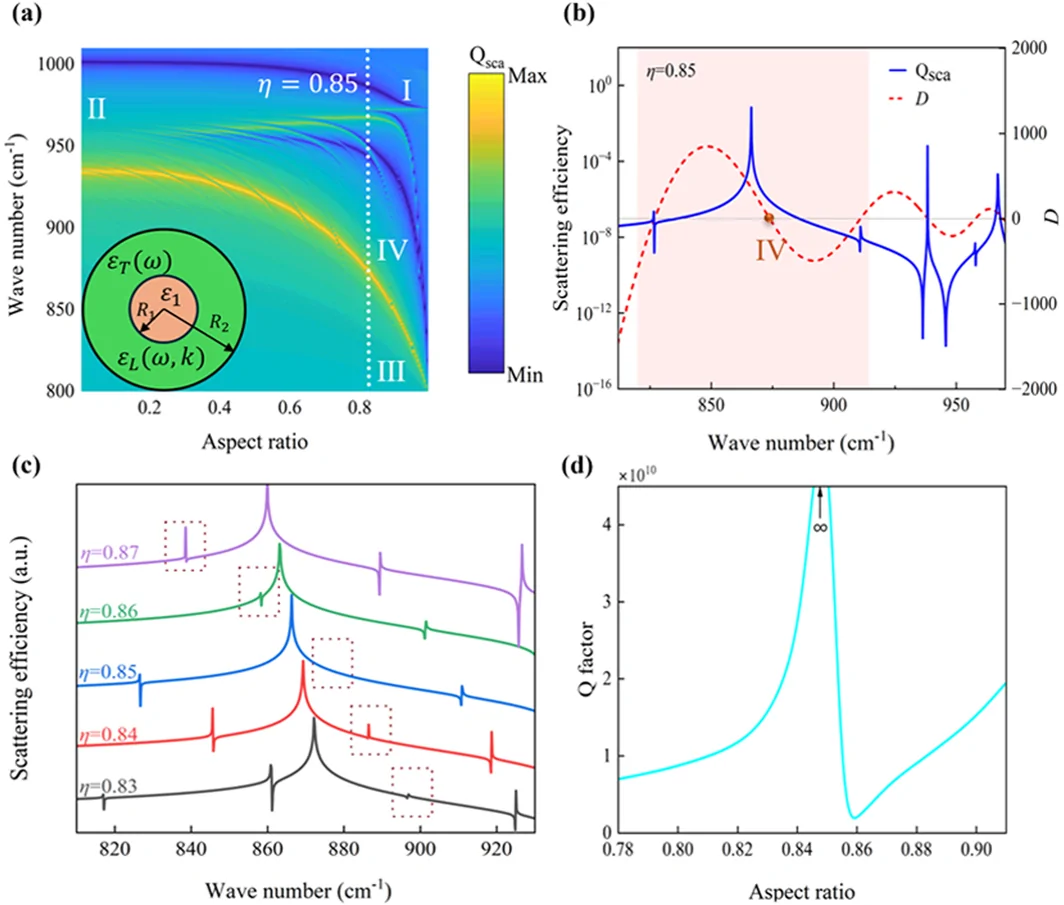
(a) Dependence of scattering efficiency $Q_{sca}$ on incident frequency ω and aspect ratio η for core–shell nanospheres under the nonlocal case. (b) Scattering efficiency (blue lines) and characteristic function D (red lines) as a function of wavenumber with $R_{1}=17\;\text{nm}$ and η=0.85, corresponding to the vertical dashed line marked in (b). (c) Scattering efficiency $Q_{sca}$ for different η, evaluated within the frequency range highlighted in the shaded region of (b). (d) Quality factor as a function of the aspect ratio η. Other parameters are the same as in Figure 2.

To clarify the formation mechanism of these multiple BICs, we analyze the scattering efficiency $Q_{sca}$ and the characteristic function $D\;\equiv \;\det\left(M_{\text{nonlocal}}\right)$ at a fixed aspect ratio η=0.85 (corresponding to the white dashed line in Figure 4a). The real‐frequency zeros of D correspond to the eigenfrequencies of the intrinsic modes of the core–shell nanoparticle. In the nonlocal case, $M_{\text{nonlocal}}$ contains contributions from LO phonon modes. Consequently, the condition D=0 admits eigenmodes with nontrivial LO phonon components inside the shell and gives rise to a standing‐wave quantization condition for the confined LO phonon modes. This quantization produces a discrete set of longitudinal eigenfrequencies.

As shown in Figure 4b, these zeros at discrete eigenfrequencies coincide precisely with the narrow Fano resonances in the scattering spectrum, demonstrating that these resonances originate from the coupling of quantized LO phonon modes to the transverse electromagnetic field. Physically, the external radiation couples directly to the transverse phonon‐polariton modes of the shell, which serve as bright states connected to the radiation continuum, whereas the quantized LO phonon modes remain confined inside the structure and are intrinsically dark. Spatial dispersion enables coupling between these transverse and longitudinal excitations, allowing the otherwise inaccessible LO phonon modes to interact indirectly with the radiation continuum. As a result, the nonresonant transverse scattering channel interferes with the resonant response of the longitudinal‐transverse hybrid modes, giving rise to the characteristic asymmetric Fano line shapes. Each quantized LO phonon mode therefore gives rise to a distinct Fano resonance.

Notably, the Fano resonance at $\omega =874{\text{cm}}^{-1}$ (point IV) vanishes where the condition D=0 is satisfied, indicating complete suppression of radiation and the emergence of a BIC [53, 54]. Although every quantized LO phonon mode produces a Fano resonance, only those whose net radiative coupling vanishes evolve into BICs. Each BIC can therefore be viewed as a longitudinal‐transverse hybrid eigenmode associated with a quantized LO phonon standing wave, whose coupling to the external radiative continuum is completely canceled through destructive interference.

To further characterize the nonlocality‐induced BIC, Figure 4c shows the evolution of a representative BIC by plotting the scattering spectrum as η increases. We see that the Fano resonance associated with the eigenfrequency of the longitudinal eigenmode undergoes a continuous redshift and linewidth narrowing as η gradually increases from 0.83. Remarkably, at the critical aspect ratio η=0.85, the Fano resonance disappears, where the scattering zero exactly overlaps the scattering resonance, indicating the emergence of a BIC. As η further increases, the Fano resonance reappears, indicating a transition back to a quasi‐BIC. Furthermore, Figure 4d presents the Q factor of the Fano resonance, showing diverging Q factor at the critical aspect ratio η=0.85, further confirming the emergence of BIC arising from locality‐associated longitudinal modes in the studied nanoparticle.

Further physical insights can be gained by analyzing the electric field distribution of the quasi‐BIC. As the aspect ratio η increases from 0.83 to 0.85 (Figure 5a–c), we see that the field distribution becomes more localized inside the particle, whereas the external field tends to vanish. Indeed, the BIC is characterized by zero field outside the nanoparticles, which indicates that the radiation damping vanishes and the Q factor tends to infinity.

**FIGURE 5:**
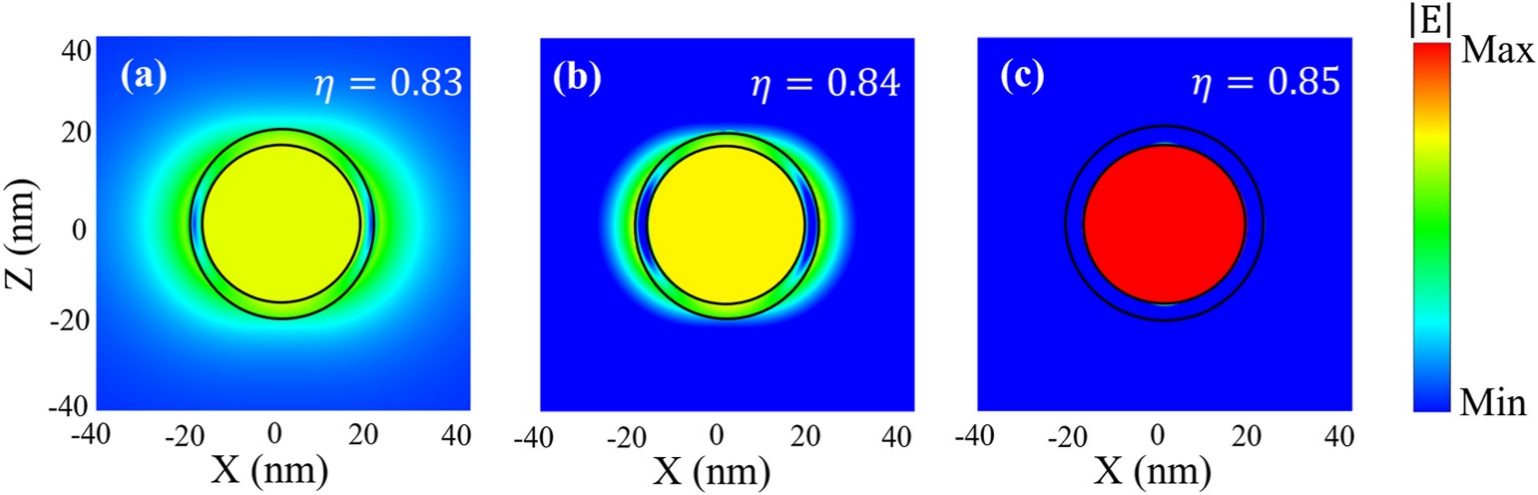
Evolution of the electric‐field amplitude distributions near BIC for different aspect ratios: (a) η=0.83, (b) η=0.84, and (c) η=0.85 under the plane‐wave illumination.

To further evaluate the robustness of the nonlocality‐induced quasi‐BIC, Figure 6 shows the dependence of the Q factor on material loss and structural perturbations at η=0.84. The circles represent the numerical results, whereas the black solid lines denote the fitted curves. All four panels share the same vertical‐axis range, allowing a direct comparison of the influence of different perturbations on the Q factor. As shown in Figure 6a, the Q factor decreases rapidly with increasing material damping coefficient γ, following approximately the scaling relation $Q\propto \gamma^{-1}$. This behavior identifies intrinsic material loss as one dominant factor limiting the Q factor of the quasi‐BIC [24, 28, 55]. By contrast, Figure 6b–d show that relative perturbations in the outer radius $\Delta R_{2}/R_{2}$ (with fixed η=0.84), the core permittivity $\Delta \varepsilon_{1}/\varepsilon_{1}$, and the background permittivity $\Delta \varepsilon_{h}/\varepsilon_{h}$ have only a minor effect on the Q factor, which remains within the same order of magnitude over the perturbation range considered. These results demonstrate that the nonlocality‐induced quasi‐BIC is considerably more sensitive to intrinsic material loss than to moderate geometrical or dielectric perturbations.

**FIGURE 6:**
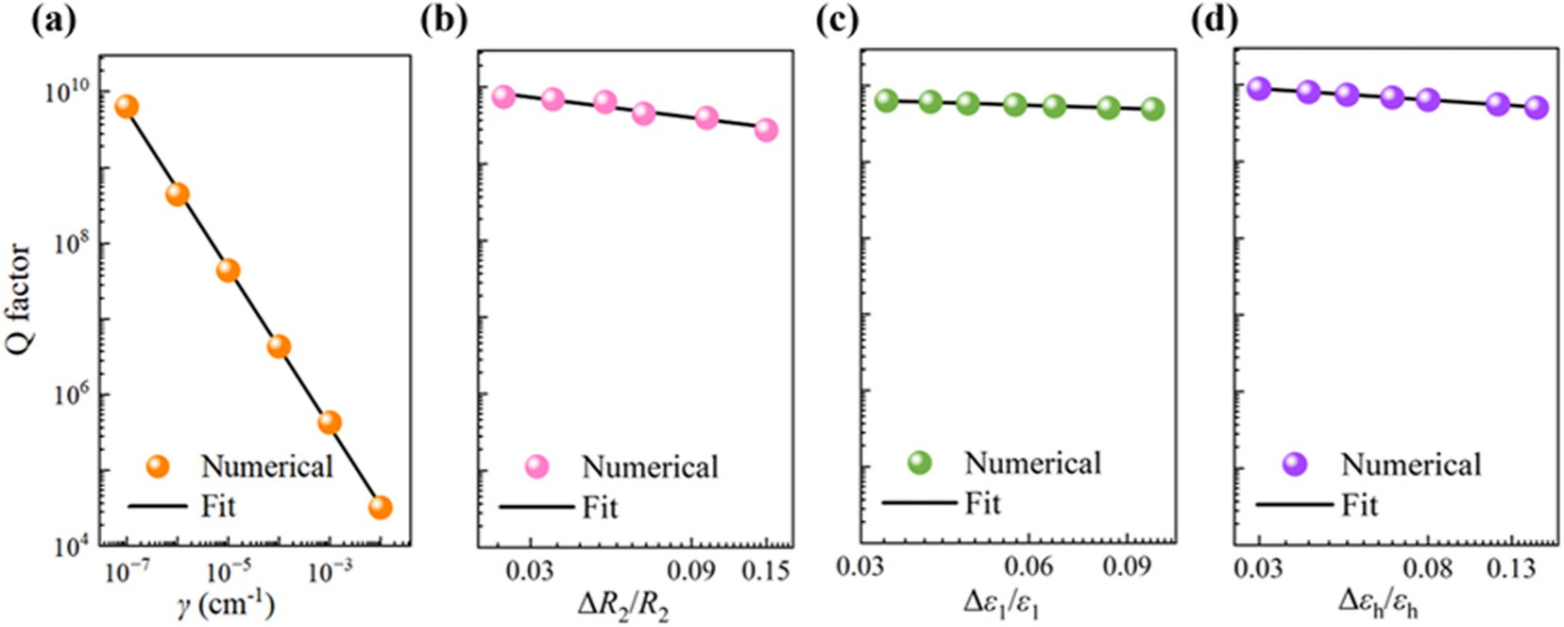
Dependence of the Q factor of the nonlocality‐induced quasi‐BIC on different perturbations at η=0.84: (a) material damping coefficient γ, (b) relative deviation of the outer radius $\Delta R_{2}/R_{2}$, relative deviation of the core permittivity $\Delta \varepsilon_{1}/\varepsilon_{1}$, and (d) relative deviation of the background permittivity $\Delta \varepsilon_{h}/\varepsilon_{h}$. Circles represent the numerical results, and black solid lines denote the fitted curves.

Although intrinsic losses and fabrication imperfections inevitably transform ideal BICs into quasi‐BICs, the underlying nonlocal physics is well supported experimentally. Hybrid longitudinal‐transverse phonon polaritons [56] have already been demonstrated in polar nanophotonic systems, providing experimental evidence for the key physical ingredients underlying the present mechanism. Therefore, while realizing the specific single‐particle geometry considered here remains technologically challenging, the predicted nonlocality‐induced quasi‐BICs could become experimentally accessible as nanofabrication and mid‐infrared characterization techniques continue to advance.

Finally, it is noteworthy that, unlike BICs in the local‐response plasmonic nanoparticles [37, 38], which emerge only under extreme material or geometrical conditions (ε=0, η→1, or η→0), the nonlocality‐induced BICs persist over a much broader region of finite parameter space. As a result, multiple BICs can be engineered within a single polar dielectric nanoparticle through dimensional tuning. In addition, the mechanism of the nonlocality‐induced BICs in polar dielectric nanoparticles differs fundamentally from that underlying BICs in dielectric supercavities [23, 28] where radiation suppression arises from destructive interference among transverse Mie‐type modes. In contrast, spatial dispersion gives rise to discrete set of LO phonon modes that couple to the transverse radiative continuum, thereby generating multiple BICs associated with distinct longitudinal radial orders.

An additional advantage of the nonlocality‐induced BICs is the extreme subwavelength confinement. These BICs can be supported in a polar dielectric nanoparticle with an outer radius of only 20 nm (∼$\lambda_{0}/500$), which is substantially smaller than the dimensions required for BICs in dielectric supercavities [23, 28] or local plasmonic nanoparticles [37, 38]. This stems from the large longitudinal wave vector of the underlying LO phonon modes (i.e., $k_{L}\gg \omega /c$), which yields characteristic wavelengths much shorter than those of the transverse modes responsible for conventional local‐response BICs. Such deeply subwavelength confinement may provide new opportunities for ultracompact nanolasers and enhanced light–matter interactions.

## 3 Conclusions

In this work, we have theoretically demonstrated that spatial dispersion in polar dielectric core–shell nanoparticles provide a powerful mechanism for achieving multiple BICs in deep‐subwavelength systems. The incorporation of nonlocality gives rise to hybrid longitudinal‐transverse modes within the polar dielectric shell. These hybrid modes induce sharp Fano resonances in the scattering spectra and, under appropriate geometric tuning, their radiative damping can be completely suppressed, leading to a series of BICs. Notably, unlike conventional BICs in local nanostructures that typically demand extreme material parameters, the nonlocality‐induced BICs demonstrated here emerge across a broad parameter space and are particularly pronounced in the deep‐subwavelength regime where discrete longitudinal modes are strongly enhanced and tightly confined. Although intrinsic material absorption may limit the achievable quality factors in practice, the relatively low‐loss nature of polar dielectrics and the possibility of gain‐assisted compensation make experimental implementation feasible. These findings establish nonlocal polar dielectric nanostructures as a versatile nanophotonic platform for enhanced light–matter interactions at the deep‐subwavelength scale with promising prospects for ultrasensitive sensing, nonlinear nanophotonics, and nanolasers.

Extending the proposed single‐particle nonlocal‐response BICs to large‐area metasurfaces represents an interesting direction for future research. Recent demonstrations of polar dielectric metasurfaces supporting symmetry‐protected BICs [21] provide a promising platform for exploring the interplay between conventional symmetry‐based BICs and nonlocality‐induced BICs. Such extensions, however, will require addressing practical challenges including substrate effects, inter‐particle coupling, finite‐size effects, and fabrication imperfections, as the nonlocality‐induced BICs are sensitive to structural perturbations and may evolve into finite‐Q quasi‐BICs. Furthermore, AI‐driven inverse design and physics‐informed machine learning could offer powerful approaches for optimizing the complex parameter space of geometry, materials, and environments, thereby improving the robustness and scalability of BIC‐based photonic architectures [57, 58].

## Author Contributions

All authors have accepted responsibility for the entire content of this manuscript and approved its submission.

## Funding

This work was supported by the National Natural Science Foundation of China (12274314, 12374293), Suzhou Basic Research Project (SJC2023003), Natural Science Foundation of Jiangsu Province (BK20233001).

## Conflicts of Interest

The authors declare no conflicts of interest.

## Data Availability

The data that support the findings of this study are available from the corresponding author upon reasonable request.
